# Effects of two amino acid substitutions in the capsid proteins on the interaction of two cell-adapted PanAsia-1 strains of foot-and-mouth disease virus serotype O with heparan sulfate receptor

**DOI:** 10.1186/1743-422X-11-132

**Published:** 2014-07-24

**Authors:** Xingwen Bai, Huifang Bao, Pinghua Li, Wei Wei, Meng Zhang, Pu Sun, Yimei Cao, Zengjun Lu, Yuanfang Fu, Baoxia Xie, Yingli Chen, Dong Li, Jianxun Luo, Zaixin Liu

**Affiliations:** 1 State Key Laboratory of Veterinary Etiological Biology, OIE/National Foot-and-Mouth Disease Reference Laboratory, Engineering Research Center of Biological Detection of Gansu Province, Lanzhou Veterinary Research Institute, Chinese Academy of Agricultural Sciences, Lanzhou, Gansu 730046, China; 2 Present Address: School of Chinese Medicine, Hong Kong Baptist University, Hong Kong Special Administrative Region, Kowloon Tong, China

**Keywords:** Foot-and-mouth disease virus, PanAsia-1 strain, Heparan sulfate receptor, Phenotypic property, Molecular determinant

## Abstract

**Background:**

Some cell-adapted strains of foot-and-mouth disease virus (FMDV) can utilize heparan sulfate (HS) as a receptor to facilitate viral infection in cultured cells. A number of independent sites on the capsid that might be involved in FMDV-HS interaction have been studied. However, the previously reported residues do not adequately explain HS-dependent infection of two cell-adapted PanAsia-1 strains (O/Tibet/CHA/6/99tc and O/Fujian/CHA/9/99tc) of FMDV serotype O.

To identify the molecular determinant(s) for the interaction of O/Tibet/CHA/6/99tc and O/Fujian/CHA/9/99tc with HS receptor, several chimeric viruses and site-directed mutants were generated by using an infectious cDNA of a non-HS-utilizing rescued virus (Cathay topotype) as the genomic backbone. Phenotypic properties of these viruses were determined by plaque assays and virus adsorption and penetration assays in cultured cells.

**Results:**

Only two of the rescued viruses encoding VP0 of O/Tibet/CHA/6/99tc or VP1 of O/Fujian/CHA/9/99tc formed plaques on wild-type Chinese hamster ovary (WT-CHO; HS+) cells, but not on HS-negative pgsD-677 cells. The formation of plaques by these two chimeric viruses on WT-CHO cells could be abolished by the introduction of single amino acid mutations Gln-2080 → Leu in VP2 of O/Tibet/CHA/6/99tc and Lys-1083 → Glu in VP1 of O/Fujian/CHA/9/99tc, respectively. Nonetheless, the introduced mutation Leu-2080 → Gln in VP2 of O/Fujian/CHA/9/99tc for the construction of expectant recombinant plasmid led to non-infectious progeny virus in baby hamster kidney 21 (BHK-21) cells, and the site-directed mutant encoding Glu-1083 → Lys in VP1 of O/Tibet/CHA/6/99tc did not acquire the ability to produce plaques on WT-CHO cells.

Significant differences in the inhibition of the infectivity of four HS-utilizing viruses by heparin and RGD-containing peptide were observed in BHK-21 cells.

Interestingly, the chimeric virus encoding VP0 of O/Fujian/CHA/9/99tc, and the site-directed mutant encoding Gln-2080 → Leu in VP2 of O/Tibet/CHA/6/99tc could bind to HS, but there was no expression of the 3A protein of these two viruses in WT-CHO cells.

**Conclusion:**

The results suggest that the cooperation of certain specific amino acid residues in the capsid proteins of these two cell-adapted PanAsia-1 strains is essential for viral infectivity, the heparin affinity and the capability on FMDV-HS interaction.

## Background

Foot-and-mouth disease (FMD) is an acute, highly contagious vesicular disease of cloven-hoofed animals including cattle, swine, sheep, and goats [1]. Its etiological agent, foot-and-mouth disease virus (FMDV), is a member of the *Aphthovirus* genus of the family *Picornaviridae*[2]. The virus genome consists of a single-stranded, positive-sense RNA molecule of approximately 8,500 nucleotides that encodes four structural viral proteins (VP1–VP4) and nine non-structural proteins (Lab/Lb, 2A, 2B, 2C, 3A, 3B_1–3_, 3C, and 3D) [3,4]. The FMDV virion has a non-enveloped, icosahedrally symmetric capsid, which is composed of 60 copies each of the surface-exposed VP1, VP2, and VP3 as well as the internal VP4 [5].

The first step during FMDV infection is the recognition of target cells by binding to cellular receptors [6]. It has been reported that FMDV could utilize four members of the αV subgroup of integrins (αVβ1, αVβ3, αVβ6, and αVβ8) as receptors in tissue culture [7-12], via a highly conserved arginine-glycine-aspartic acid (RGD) motif located within the G-H loop of VP1 [8,13-15]. In addition to integrins, FMDV likewise utilizes multiple membrane molecules as receptors. These alternative receptors include heparan sulfate (HS) [16], the Fc receptor (FcR) [15,17], the synthetic scab/ICAM1 protein (a FMDV-specific, single-chain monoclonal antibody fused to the intercellular adhesion molecule 1) [18], and certain still unknown molecules on the cell surface that are neither integrins nor HS [19-24].

HS is a glycosaminoglycan polymer of disaccharide repeats of L-iduronic acid (Idu) and D-glucosamine (GlcN), which is highly sulfated and thus negatively charged [25,26]. The ability of FMDV to utilize HS as a receptor enables the virus to grow more efficiently in cultured cells, whereas viruses that have a high affinity for heparin (one of HS-like substances) are attenuated for cattle [27]. Studies of FMDV-HS interaction have identified the acquisition of positively charged residues during HS adaptation at widely spaced locations on the outer capsid surface of FMDV serotypes O [16,24,27,28], A [24,29], C [20-22], SAT1 [30,31], and SAT2 [30]. Here, several chimeric viruses and site-directed mutants were generated to identify the molecular determinant(s) of the HS-binding that contribute to establishing an efficient infection by two cell-adapted PanAsia-1 viruses in wild-type Chinese hamster ovary (WT-CHO) cells. Furthermore, phenotypic properties of these viruses were explored with focus on the particular effect of the addition of heparin and RGD-containing peptide in FMDV infection of baby hamster kidney 21 (BHK-21) cells and the potential consequences of the indicated FMDVs to bind the cell surface HS of WT-CHO cells. Our results are useful for improving present knowledge of the molecular basis of FMDV-HS interaction in cultured cells.

## Results

### Plaque phenotypes of the selected Cathay topotype and PanAsia-1 strains of FMDV serotype O

**Figure 1:**
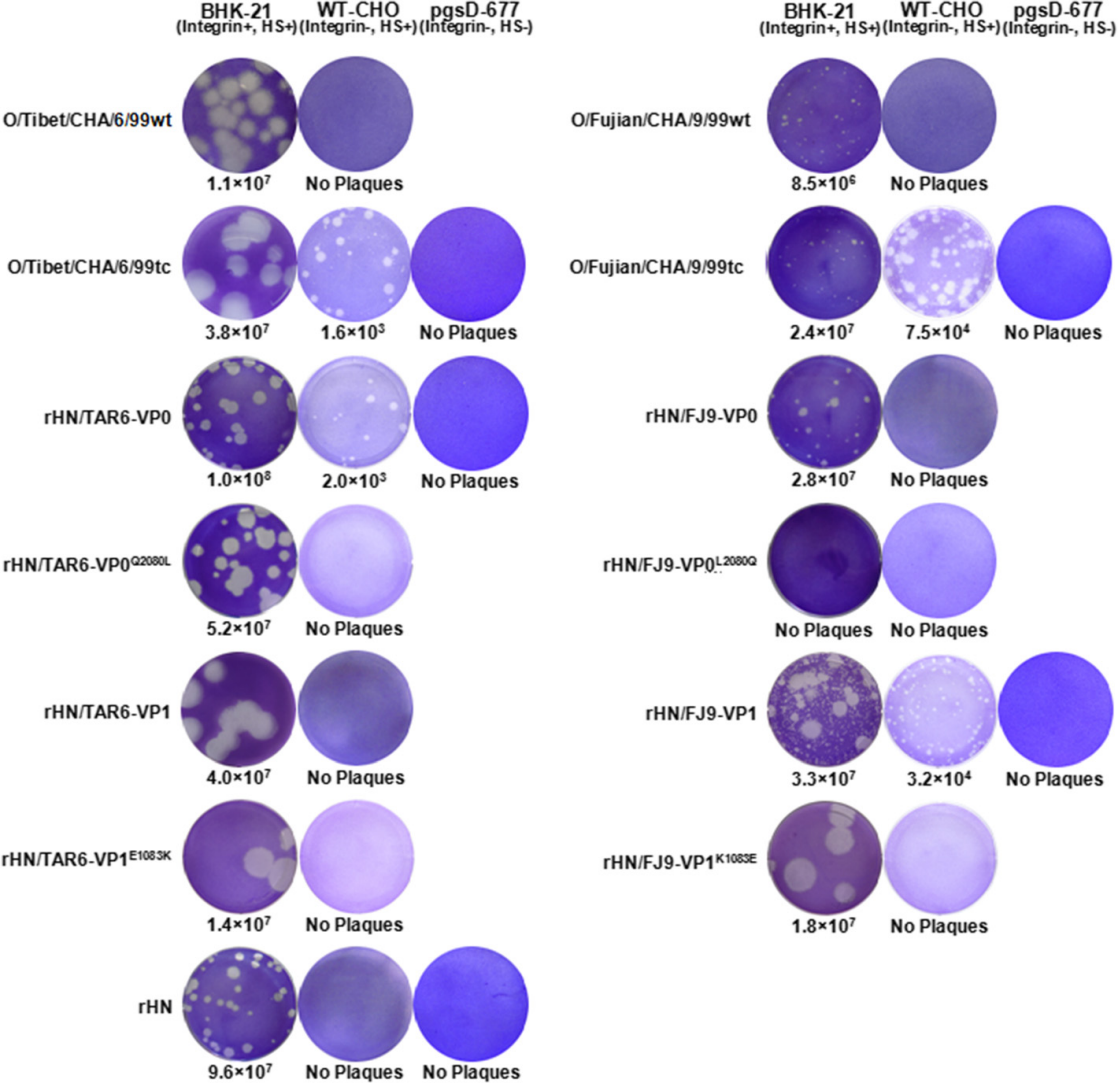
**Plaque phenotypes of all the selected PanAsia-1 strains and rescued FMDVs on BHK-21 cells and two CHO cell lines.** Subconfluent monolayers of cultured cells infected with the specific viruses were stained with 0.2% crystal violet at 48 h post-infection (BHK-21 cells) and 72 h post-infection (WT-CHO and pgsD-677 cells). Virus titres (PFU/mL) are listed at the bottom of each image. The infection of rHN/FJ9-VP0^L2080Q^ collected from the supernatant of pOFS/FJ9-VP0^L2080Q^ (see Figure 2) co-transfected with pCT7RNAP after 48 h was not detected in BHK-21 cells as determined by the plaque forming assay. Only two cell-adapted PanAsia-1 viruses (O/Tibet/CHA/6/99tc and O/Fujian/CHA/9/99tc) and two chimeric viruses (rHN/TAR6-VP0 and rHN/FJ9-VP1) formed plaques on WT-CHO cells.

For our initial studies, we performed plaque forming assay of a genetically engineered virus rHN (Cathay topotype, recovered from an infectious cDNA pOFS), two bovine-isolated (O/Tibet/CHA/6/99wt and O/Fujian/CHA/9/99wt; wild-type) and their cell-adapted (O/Tibet/CHA/6/99tc and O/Fujian/CHA/9/99tc; tissue culture) PanAsia-1 viruses on WT-CHO and HS-negative pgsD-677 cells. Comparative analysis showed that rHN, O/Tibet/CHA/6/99wt and O/Fujian/CHA/9/99wt were unable to produce plaques on WT-CHO cells (Figure 1). However, O/Tibet/CHA/6/99tc and O/Fujian/CHA/9/99tc formed plaques on WT-CHO cells, but not on pgsD-677 cells (Figure 1). These results demonstrated that (i) rHN, O/Tibet/CHA/6/99wt and O/Fujian/CHA/9/99wt are unable to utilize HS as a receptor for viral infection in WT-CHO cells; (ii) O/Tibet/CHA/6/99tc and O/Fujian/CHA/9/99tc have acquired the HS-utilizing ability to establish an efficient infection in WT-CHO cells.

### Molecular characterization of the capsid proteins of the field-isolated and cell-adapted PanAsia-1 FMDVs

To map amino acid differences in the capsid proteins between O/Tibet/CHA/6/99wt and O/Tibet/CHA/6/99tc, O/Fujian/CHA/9/99wt and O/Fujian/CHA/9/99tc, nucleotide sequences of the capsid coding regions of these PanAsia-1 viruses were determined. The deduced amino acid sequences of O/Tibet/CHA/6/99tc and O/Fujian/CHA/9/99tc were compared with that of O/Tibet/CHA/6/99wt and O/Fujian/CHA/9/99wt, respectively. Alignment of VP1–VP4 coding sequences displayed that amino acid substitutions were presented in VP4 (Ala-4008 → Ser), VP2 (Leu-2080 → Gln and Glu-2136 → Gly) and VP1 (Val-1144 → Ala, Thr-1158 → Ala, and Leu-1212 → Ser) of O/Tibet/CHA/6/99tc as well as in VP2 (Arg-2056 → His) and VP1 (Lys-1041 → Thr, Lys-1045 → Gln, Glu-1083 → Lys, Glu-1095 → Ala, and Ser-1139 → Arg) of O/Fujian/CHA/9/99tc (Table 1). Of the 12 different amino acid changes (including Ala-4008 → Ser in VP4 of O/Tibet/CHA/6/99tc), only two positively charged amino acid residues were found in the capsid protein VP1 (Glu-1083 → Lys and Ser-1139 → Arg) of O/Fujian/CHA/9/99tc (Table 1). The deduced amino acid sequences of the VP3 coding region of O/Tibet/CHA/6/99tc and O/Fujian/CHA/9/99tc were 100% identical to O/Tibet/CHA/6/99wt and O/Fujian/CHA/9/99wt, respectively. It thus appears that the specific amino acid residues on the outer capsid proteins VP2 and VP1 of O/Tibet/CHA/6/99tc (neutral amino acid residues) and O/Fujian/CHA/9/99tc may be necessary for FMDV-HS interaction.

**Table 1:** Amino acid differences in the capsid proteins of the selected PanAsia-1 strains of FMDV serotype O

**Capsid protein**	Position*	**Amino acid differences**^**§**^				**Secondary structure**^**£**^
		**O/Tibet/CHA/6/99wt**	**O/Tibet/CHA/6/99tc**	**O/Fujian/CHA/9/99wt**	**O/Fujian/CHA/9/99tc**	
VP4	4008	Ala (A)	**Ser (S)**	Ala (A)	Ala (A)	N-terminus
VP2	2065	His (H)	His (H)	**Arg (R)**	His (H)	B-B knob
	2079	Tyr (Y)	Tyr (Y)	***His (H)***	***His (H)***	B-C loop, close to antigenic site 2
	2080	Leu (L)	**Gln (Q)**	Leu (L)	Leu (L)	
	2136	Glu (E)	**Gly (G)**	Glu (E)	Glu (E)	E-F loop, close to antigenic site 2
VP1	1041	Lys (K)	Lys (K)	Lys (K)	**Thr (T)**	B-C loop, antigenic site 3
	1045	Lys (K)	Lys (K)	Lys (K)	**Gln (Q)**	
	1083	Glu (E)	Glu (E)	Glu (E)	**Lys (K)**	D-E loop
	1095	Glu (E)	Glu (E)	Glu (E)	**Ala (A)**	E-F loop
	1139	Ser (S)	Ser (S)	Ser (S)	**Arg (R)**	G-H loop, close to antigenic site 1
	1144	Val (V)	**Ala (A)**	Val (V)	Val (V)	G-H loop, antigenic site 1
	1158	Thr (T)	**Ala (A)**	Thr (T)	Thr (T)	
	1212	Leu (L)	**Ser (S)**	Leu (L)	Leu (L)	C-terminus, antigenic site 1

*, Amino acid residues are denoted by a standard four-digit numbering system. The first digit represents the protein (VP4, VP2 or VP1) and the remaining three digits the amino acid position, numbered independently for each protein from the amino (N-) terminus to the carboxyl (C-) terminus.

^§^, Three-letter amino acid codes are used (one-letter amino acid code is given in parentheses). The specific amino acid residues in the viral capsid proteins are shown in bold. His-2079 in O/Fujian/CHA/9/99wt and O/Fujian/CHA/9/99tc (bold and italics) is different from Tyr-2079 in the majority of sequences in the alignment of VP2 of PanAsia-1 viruses.

^£^, secondary structure assignments are as described in references 39, 40 and references cited in 46.

### Identification of the molecular determinant(s) for the interaction of two HS-utilizing PanAsia-1 FMDVs with HS receptor

**Figure 2:**
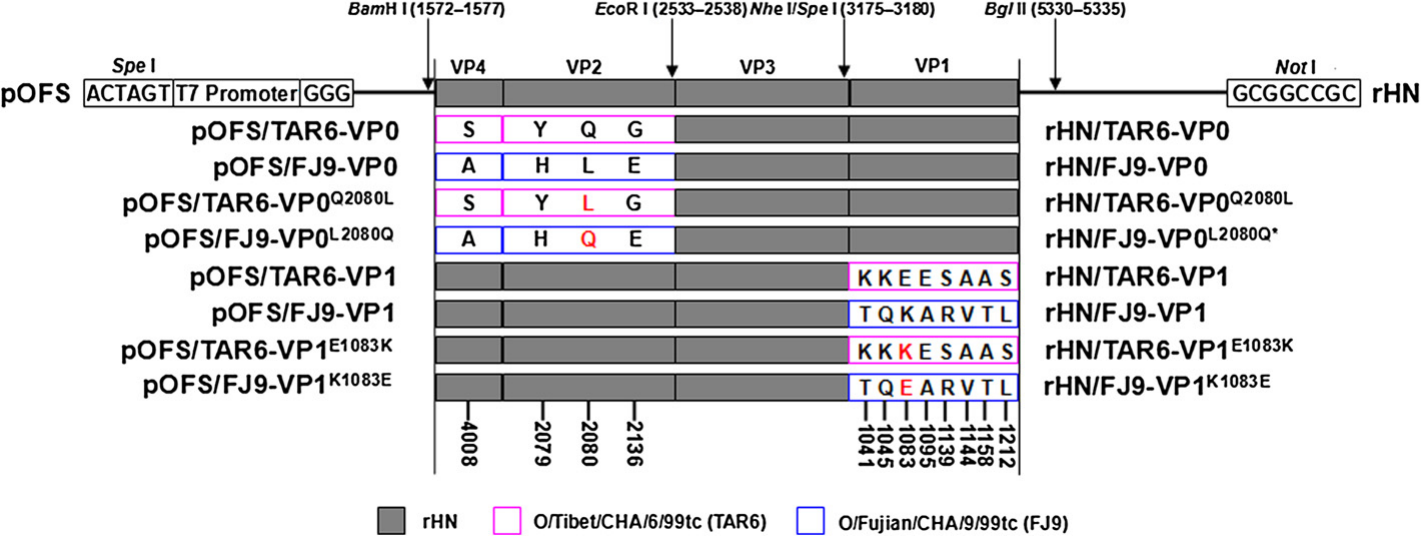
**Schematic representation of the capsid coding regions of the chimeric and site-directed mutated full-length genomic cDNA clones.** The parental plasmid pOFS of rHN was used as the genomic backbone [42] to generate the chimeric and site-directed mutated full-length cDNAs (left) of each rescued virus (right). The VP1–VP4 coding regions of rHN, O/Tibet/CHA/6/99tc (TAR6) and O/Fujian/CHA/9/99tc (FJ9) are represented by solid grey boxes with black borders, and hollow boxes with purple and blue borders, respectively. One-letter amino acid codes are used and positions at which amino acid residues differ in the compared chimeric and site-directed mutated regions are labeled at the bottom (see Table 1). Single amino acid substitutions at residues 2080 of the VP2 coding region and 1083 of the VP1 coding region are marked in red. Procedures for the construction of chimeric and site-directed mutated full-length cDNA clones are described in Methods. *, The site-directed mutant rHN/FJ9-VP0^L2080Q^ could not be rescued successfully from the original plasmid pOFS/FJ9-VP0^L2080Q^ in BHK-21 cells (see Figure 1).

To identify the functional regions of O/Tibet/CHA/6/99tc and O/Fujian/CHA/9/99tc that are responsible for FMDV-HS interaction to establish an efficient infection in WT-CHO cells, the coding regions of VP0 (VP4 and VP2) and VP1 genes of O/Tibet/CHA/6/99tc (TAR6) and O/Fujian/CHA/9/99tc (FJ9) were substituted into plasmid pOFS to construct the chimeric full-length cDNA clones, pOFS/TAR6-VP0, pOFS/TAR6-VP1, pOFS/FJ9-VP0 and pOFS/FJ9-VP1, respectively (Figure 2). Four chimeric viruses were rescued from the respective *Not* I-linearized plasmid constructs co-transfected with pCT7RNAP in BHK-21 cells, and designated as rHN/TAR6-VP0, rHN/TAR6-VP1, rHN/FJ9-VP0, and rHN/FJ9-VP1. In the plaque forming assay, rHN/TAR6-VP0 and rHN/FJ9-VP1 formed plaques on WT-CHO cells, whereas none were produced on pgsD-677 cells (Figure 1). By contrast, rHN/FJ9-VP0 and rHN/TAR6-VP1 were unable to produce plaques on WT-CHO cells (Figure 1). Hence, it follows that a few specific amino acid residues in VP2 of O/Tibet/CHA/6/99tc (Gln-2080 and/or Gly-2136) and VP1 of O/Fujian/CHA/9/99tc (especially Lys-1083 and/or Arg-1139) may play a more important role in the interaction of rHN/TAR6-VP0 and rHN/FJ9-VP1 with the HS receptor, respectively.

As shown by previous studies, the similarities in the crystal structures of the FMDV-HS complexes (serotype O and A) suggest that this binding site has an important role in the survival of the virus, perhaps via persistent infections [28,29,32]. A substitution change at position 2080 in VP2 (Ala to Gln) of UKG/34/2001 (PanAsia-1 strain) has been observed from persistently infected cattle [33]. Furthermore, a cell culture adaptation of O/CHA/90 (Cathay topotype) had acquired the ability to grow in WT-CHO cells via the accumulation of a positively charged residue (Glu to Lys) at position 1083 of VP1 [34]. To further investigate the molecular basis of the ability of rHN/TAR6-VP0 and rHN/FJ9-VP1 to utilize the HS receptor for efficient infection in WT-CHO cells, single amino acid mutations Gln-2080 → Leu in VP2 of O/Tibet/CHA/6/99tc and Lys-1083 → Glu in VP1 of O/Fujian/CHA/9/99tc were introduced into pOFS/TAR6-VP0 and pOFS/FJ9-VP1, respectively, to construct two site-directed mutated cDNA clones (pOFS/TAR6-VP0^Q2080L^ and pOFS/FJ9-VP1^K1083E^) (Figure 2). Two site-directed mutants (rHN/TAR6-VP0^Q2080L^ and rHN/FJ9-VP1^K1083E^) were rescued from pOFS/TAR6-VP0^Q2080L^ and pOFS/FJ9-VP1^K1083E^ in BHK-21 cells and, as expected, rHN/TAR6-VP0^Q2080L^ and rHN/FJ9-VP1^K1083E^ lost the ability to form plaques on WT-CHO cells (Figure 1). Subsequently, two expectant recombinant plasmids (pOFS/FJ9-VP0^L2080Q^ and pOFS/TAR6-VP1^E1083K^) were constructed with the introduction of single amino acid mutations Leu-2080 → Gln in VP2 of O/Fujian/CHA/9/99tc and Glu-1083 → Lys in VP1 of O/Tibet/CHA/6/99tc into pOFS/FJ9-VP0 and pOFS/TAR6-VP1, respectively (Figure 2), to verify if these two site-directed mutants (rHN/FJ9-VP0^L2080Q^ and rHN/TAR6-VP1^E1083K^) can be generated from BHK-21 cells and initiate HS-dependent infection in WT-CHO cells. Unfortunately, rHN/FJ9-VP0^L2080Q^ was not rescued successfully from pOFS/FJ9-VP0^L2080Q^ following co-transfection in BHK-21 cells (Figure 1; no detectable RT-PCR products, result not shown). rHN/TAR6-VP1^E1083K^, the site-directed mutant rescued from pOFS/TAR6-VP1^E1083K^, did not acquire the ability to produce plaques on WT-CHO cells (Figure 1). Therefore, these results suggested that (i) single amino acid mutations in the capsid proteins VP2 of O/Tibet/CHA/6/99tc (Gln-2080 → Leu) and VP1 of O/Fujian/CHA/9/99tc (Lys-1083 → Glu) can abolish the HS-utilizing ability of the chimeric viruses (rHN/TAR6-VP0 and rHN/FJ9-VP1) to infect WT-CHO cells; (ii) some of the different amino acids in VP0 of O/Fujian/CHA/9/99tc, as compared to O/Tibet/CHA/6/99tc, may have an influence on the infectivity of the construction of a site-directed mutated cDNA clone (pOFS/FJ9-VP0^L2080Q^) in BHK-21 cells; and (iii) the incapability of the site-directed mutant (rHN/TAR6-VP1^E1083K^) to utilize HS receptor on the surface of WT-CHO cells is probably related to the differences of certain specific amino acid residues in VP1 between O/Tibet/CHA/6/99tc and O/Fujian/CHA/9/99tc (see more details in Discussion).

### Inhibition of FMDV infection by heparin and RGD-containing peptide in cultured cells

**Figure 3:**
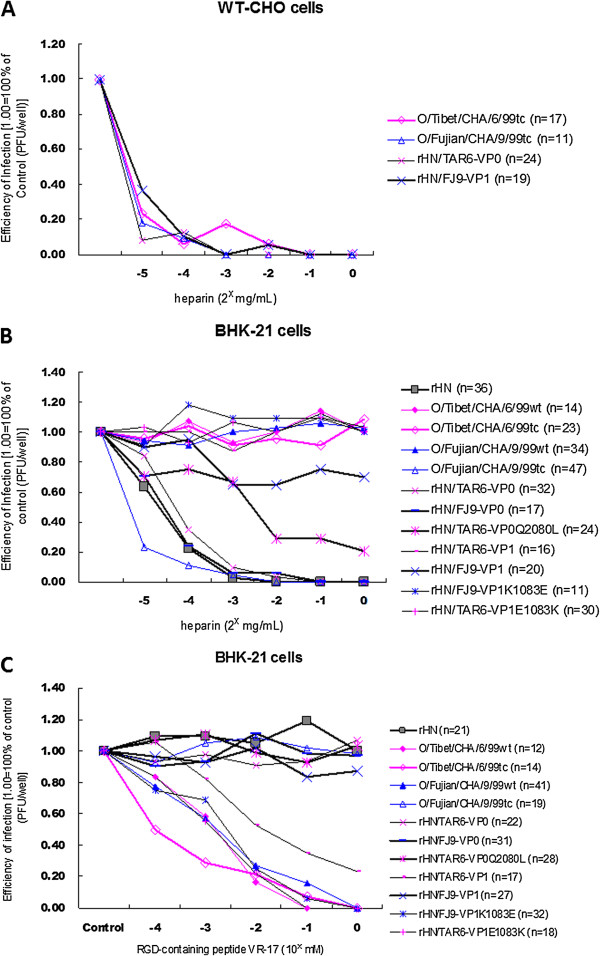
**Effects of heparin and the RGD-containing peptide on the plaque formation by the specific FMDVs on WT-CHO cells and BHK-21 cells.** The plaque reduction neutralization assay was performed on two different cell types and the plaque inhibition assay was performed on BHK-21 cells, following the procedures as described in Methods. *n* = the number of 100% PFU (10–50 plaques/well) formed by the appropriate dilutions of the indicated FMDVs. **(A)** Heparin treatment greatly reduced production of plaques with four HS-utilizing viruses on WT-CHO cells. **(B)** The numbers of plaques of rHN, rHN/FJ9-VP0 and rHN/TAR6-VP0^Q2080L^ were significantly reduced on BHK-21 cells, by addition of heparin; and **(C)** pre-incubation of the RGD-containing peptide VR-17 inhibited the plaque formation of O/Tibet/CHA/6/99tc on BHK-21 cells, despite the distinct plaque phenotypes of these four viruses on WT-CHO cells.

A plaque reduction neutralization assay was employed to measure the effectiveness of the heparin affinity of four HS-utilizing viruses on WT-CHO cells and all the selected PanAsia-1 strains and rescued viruses on BHK-21 cells. As shown in Figure 3A, the formation of plaques by O/Tibet/CHA/6/99tc, O/Fujian/CHA/9/99tc, rHN/TAR6-VP0 and rHN/FJ9-VP1 was effectively inhibited on WT-CHO cells with the addition of higher concentrations (≥0.5 mg/mL) of heparin to the diluted supernatants of each virus. The data also provided the evidence for HS-dependent infection of these four viruses in WT-CHO cells.

Heparin treatment (concentrations from 0 mg/mL to 1 mg/mL) had no effect on the plaque formation of O/Tibet/CHA/6/99wt, O/Fujian/CHA/9/99wt, rHN/TAR6-VP1, rHN/FJ9-VP1^K1083E^ and rHN/TAR6-VP1^E1083K^. It greatly reduced the production of plaques with O/Fujian/CHA/9/99tc and rHN/TAR6-VP0, and partially reduced the numbers of plaques produced by rHN/FJ9-VP1 on BHK-21 cells (Figure 3B). However, there was no detectable inhibition in the plaque formation of O/Tibet/CHA/6/99tc by heparin on BHK-21 cells (Figure 3B). As a matter of fact, the most interesting finding is that heparin was a strong inhibitor of the plaque formation on BHK-21 cells by rHN, rHN/FJ9-VP0 and rHN/TAR6-VP0^Q2080L^ (Figure 3B).

We also examined the capacity of the RGD-containing peptide VR-17 to inhibit the infectivity of the selected FMDVs by using plaque inhibition assay on BHK-21 cells, and the inhibition of FMDV infectivity by the RGD-containing peptide VR-17 further corroborated the result from the plaque reduction neutralization assay (Figure 3C). Taken together, the complementary data revealed that (i) O/Tibet/CHA/6/99wt, O/Fujian/CHA/9/99wt, rHN/TAR6-VP1, rHN/FJ9-VP1^K1083E^ and rHN/TAR6-VP1^E1083K^ utilize an integrin-mediated pathway for viral infection in BHK-21 cells; (ii) O/Fujian/CHA/9/99tc and rHN/TAR6-VP0 can completely dispense with their RGD integrin-binding site and utilize heparin-sensitive receptor(s) to infect BHK-21 cells; and (iii) efficient infection with rHN/FJ9-VP1 should be mediated by the presence of integrins and heparin-sensitive receptors on the surface of BHK-21 cells. Besides that, it seems that (i) the infection of BHK-21 cells by O/Tibet/CHA/6/99tc is not necessarily mediated by the presence of HS on the cell surface, even though this virus would be able to initiate HS-dependent infection in WT-CHO cells and (ii) rHN, rHN/FJ9-VP0 and rHN/TAR6-VP0^Q2080L^ may possibly bind to the cell surface HS, even though none of these three viruses induced plaques on WT-CHO cells (Figure 1).

### Detection of the binding of the indicated FMDVs to HS on the surface of WT-CHO cells

**Figure 4:**
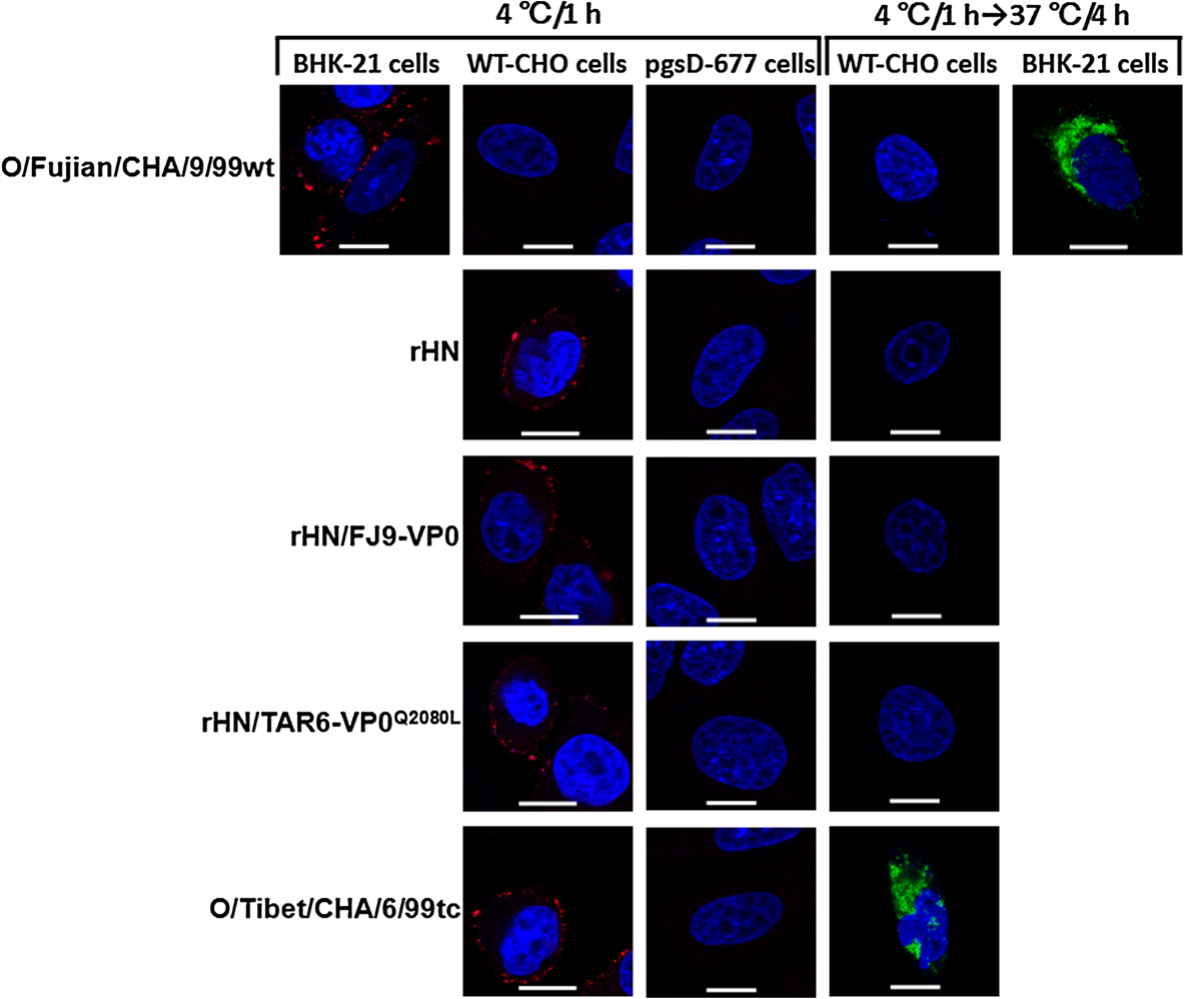
**Analysis of the interaction of the indicated FMDVs with the cell surface HS of WT-CHO cells by IFA.** In virus adsorption assay, WT-CHO cells were inoculated with O/Fujian/CHA/9/99wt and pgsD-677 cells were inoculated with each of the indicated virus as a negative control and references for non-HS binding, respectively. BHK-21 cells infected with O/Fujian/CHA/9/99wt were used as a parallel comparison (positive controls) in virus adsorption and penetration assays. Only the merged photomicrographs are shown (bar = 10 μm). The HS-binding of O/Tibet/CHA/6/99tc, rHN, rHN/FJ9-VP0 and rHN/TAR6-VP0^Q2080L^ (red) and the synthesis of 3A protein of O/Tibet/CHA/6/99tc (green) were viewed by confocal microscope.

To determine the interaction of rHN, O/Tibet/CHA/6/99tc, rHN/FJ9-VP0 and rHN/TAR6-VP0^Q2080L^ with HS on the cell surface, virus adsorption and penetration assays were performed in cultured cells. At the appropriate times, the FMDV-inoculated cells were probed for the virions or nonstructural protein 3A as described in Methods. Antibody-complexed FMDVs (red) were viewed on the surface of WT-CHO cells but not pgsD677 cells, and synthesis of the 3A protein (green) of O/Tibet/CHA/6/99tc, but not rHN, rHN/FJ9-VP0 and rHN/TAR6-VP0^Q2080L^, was viewed in WT-CHO cells (Figure 4). From the above, these results illustrated that the HS receptor is required for viral infection of WT-CHO cells by O/Tibet/CHA/6/99tc and the HS-binding of rHN, rHN/FJ9-VP0 and rHN/TAR6-VP0^Q2080L^ may not be sufficient to establish an efficient infection in WT-CHO cells.

## Discussion

The interaction of FMDV with HS has been discussed by structural studies of two tissue culture-adapted strains of FMDV (O_1_BFS and A10_61_) in a complex with heparin [28,29]. The HS-binding sites (Lys-2134, Arg-2135 and Tyr-2138 in VP2; Arg-3056, Gly-3059, Gly-3060, Ser-3087 and Asn-3088; and His-1195 in VP1) occupy critical positions that act as ligands for the five sugar residues Idu 1 to 5 and come into contact with each other [28,32]. The amino acid residues at positions 3056 (Arg) and 3084 (Lys) are involved in heparin binding of A10_61_[29], and a single amino acid change Arg-3056 → His is critical to modulate the affinity of O_1_BFS and A10_61_ to heparin [28]. Arg-2135 in VP2 and Arg-3056 in VP3 were conserved between the serotype O and A FMDV-heparin complexes. Arg-2135 of VP2 makes a hydrophobic interaction in both complexes and appears to polarize Asn-3088 of VP3, increasing its affinity for GlcN-4-6-O-SO_3_[29]. Some experimental evidence suggests that the alteration in the HS-binding may be mediated by only one or two amino acid changes in the three surface-exposed capsid proteins of FMDV [20-22,24,27,30]. Sa-Carvalho *et al*. have reported that the positively charged residues at both 2134 (Lys) in VP2 and 3056 (Arg) in VP3 were required for the binding of FMDV O_1_Campos to heparin [27]. Amino acid substitutions were found in the HS-adapted SIR (soluble integrin resistant) isolates of A_24_ Cruzeiro (A_24_-SIR#45) and O_1_Campos (O_1_C-SIR#46): Leu-1150 → Arg (RGD + 4) in VP1 of A_24_-SIR#45, His-3056 → Arg and Asp-3060 → Ala in VP3 and Glu-1113 → Val in VP1 of O_1_C-SIR#46 [24]. The selected variants of C_3_Arg/85 (C_3_-Rb) had acquired the ability to bind heparin and to infect WT-CHO cells, and Asp-3009 → Ala in VP3 and either Gly-1110 → Arg or His-1108 → Arg in VP1 were located at the five fold axis of the viral capsid [21]. The positively charged Lys-3173 in VP3 (the same position at 3174 in VP3 of O_1_BFS) is essential and Ser-1144 (RGD + 1) in VP1 may also play a role for the binding of MARLS (selected from C-S8c1) to heparin [20]. Loss of heparin binding in C-S8c1p100c10 was associated with a single amino acid replacement which was either Lys-3173 → Glu in VP3 or Arg-1197 → His (the same position at 1195 in VP1 of O_1_BFS) at the C-terminal region of VP1 [22]. In serotype SAT1 FMDVs, a recombinant virus vSAT1tc (derived from SAT1/SAR/9/81tc) displayed a high affinity for WT-CHO cells and the VP1 Lys-1110 → Asn replacement could abrogate the ability to infect WT-CHO cells [31]. The introduction of amino acid mutations at position Gly-1111 → Arg and Gly-1112 → Arg in VP1 of cell culture-adapted SAT1/NAM/307/98 (vNAM(KRR)/SAT) is thought to correlate with the acquisition of the HS-utilizing ability for viral infection of WT-CHO cells [30]. Thus, these implied that FMDV has a high heparin affinity, which is accompanied by the fixation of not only positively charged residues but also neutral amino acid residues surrounding the G-H loop of VP1 and the fivefold axis of the virus. However, no amino acid differences at these potential HS-binding sites were found on the capsid of O/Tibet/CHA/6/99tc and O/Fujian/CHA/9/99tc, as compared to their non-HS-adapted field isolates (O/Tibet/CHA/6/99wt and O/Fujian/CHA/9/99wt), respectively. All the selected PanAsia-1 viruses carried Lys-2134, Arg-2135 and Tyr-2138 in VP2, Asp-3009, His-3056, Gly-3059, Asp-3060, Lys-3084, Ser-3087, Asn-3088 and Ala-3174 in VP3, and His-1108, Ala-1110, Pro-1111, Leu-1112, Thr-1113, Leu-1148 (RGD + 1), Leu-1151 (RGD + 4) and His-1195 in VP1. The neutral amino acid change Ala-1144 (substituted for Val) was located at position RGD-1 in VP1 of O/Tibet/CHA/6/99tc (Table 1), and the chimeric virus encoding VP1 of O/Tibet/CHA/6/99tc (rHN/TAR6-VP1) did not exhibit more efficient binding to HS for viral infection in WT-CHO cells (Figure 1).

**Figure 5:**
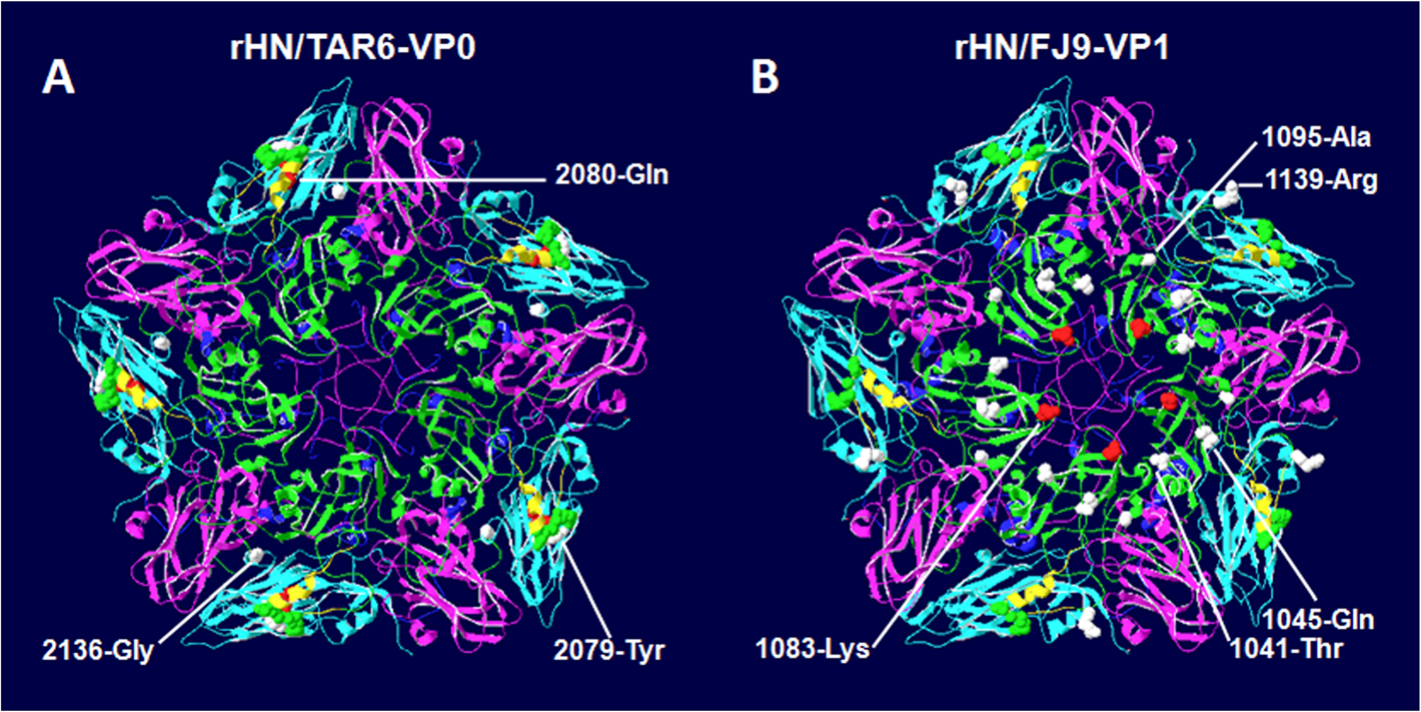
**Locations of the distinct amino acid residues in the FMDV capsid of (A) rHN/TAR6-VP0 and (B) rHN/FJ9-VP1.** The crystallographic coordinates of O1BFS (missing the first 15 amino acid residues at the N-terminus of VP4) were used as templates [35], and 3D structures of one of the twelve pentamers were optimized using the SWISS-MODEL program [36-38]. The respective capsid proteins VP1–4 are represented as green, cyan, magenta, and blue (internal). The G-H loop of VP1 is highlighted in yellow. The RGD motif (residues 1145–1147, colored green), residues 2079, 2080 (colored red) and 2136 in VP2 of O/Tibet/CHA/6/99tc, as well as residues 1041, 1045, 1083 (colored red), 1095 and 1139 in VP1 of O/Fujian/CHA/9/99tc are displayed in the space-filling models.

Nonetheless, the 3D structural models of rHN/TAR6-VP0 and rHN/FJ9-VP1 indicated that Gln-2080 in the B-C loop of VP2 of O/Tibet/CHA/6/99tc and Lys-1083 within the D-E loop of VP1 of O/Fujian/CHA/9/99tc surround the G-H loop of VP1 and the pore at the fivefold axis of symmetry, respectively (Figure 5A, 5B) [35-38]. The difference in phenotypic properties of the two chimeric viruses (rHN/TAR6-VP0 and rHN/FJ9-VP1) and their site-directed mutants (rHN/TAR6-VP0^Q2080L^ and rHN/FJ9-VP1^K1083E^) on WT-CHO cells were consistent with our expectations (Figure 1). Yet, the introduction of a Leu-2080 → Gln mutation in VP2 of O/Fujian/CHA/9/99tc (pOFS/FJ9-VP0^L2080Q^) was particularly deleterious for the generation of progeny virus in BHK-21 cells, and a Glu-1083 → Lys mutation in VP1 of O/Tibet/CHA/6/99tc did not result in the acquisition of the HS-utilizing ability of rHN/TAR6-VP1^E1083K^ to infect WT-CHO cells (Figure 1). Moreover, a second-site mutation (Glu-2136 → Gly) in VP2 of O/Fujian/CHA/9/99tc was unable to compensate for the lethal effect of the primary mutation Leu-2080 → Gln in the VP2 coding region of pOFS/FJ9-VP0^L2080Q^ and the site-directed mutant encoding Glu-1083 → Lys and Ser-1139 → Arg in VP1 of O/Tibet/CHA/6/99tc also could not utilize HS as a receptor to establish an efficient infection in WT-CHO cells (results not shown). There are a limited number of amino acid differences fixed in the VP2 and VP1 coding regions of the mutated plasmids as compared to rHN/TAR6-VP0 and rHN/FJ9-VP1, respectively (Table 1). In particular, the distinct amino acid residues at 2079 (Tyr) of VP2 lies adjacent to the RGD motif (Figure 5A), and (ii) Thr-1041 and Gln-1045 of VP1 are clustered around the fivefold axis (Figure 5B). The outcome of the substitution at residue 2079 of VP2 could potentially dislocate both antigenic sites 1 (1141–1160 residues of the VP1 G-H loop, [39]) and 2 (2070–2078 residues of the VP2 B-C loop, [40]), with a structural and/or functional change [33]. One of the amino acid mutations, Lys-1041 → Glu in VP1 was fixed in two heparin-binding derivatives of C-S8c1 (C-S8c1p100c10 and the MARLS mutant) [20,22]. Accordingly, His-2079 → Tyr in VP2 of O/Fujian/CHA/9/99tc and Lys-1141 → Thr and/or Lys-1145 → Gln in VP1 of O/Tibet/CHA/6/99tc may potentially be able to restore infectious viral phenotypes in BHK-21 cells, and be helpful in gaining the HS-utilizing ability of the corresponding site-directed mutants for viral infection in WT-CHO cells. In other words, the specific amino acid residues at positions 1041 (Lys) and 1045 (Lys) in VP1 of O/Tibet/CHA/6/99tc and 2079 (His) in VP2 of O/Fujian/CHA/9/99tc (Table 1), could be detrimental to the genetic response of the indicated viruses to FMDV-HS interaction.

It should be noted especially that the acquisition of the ability to infect WT-CHO cells as a result of substitutions at the functionally critical residues in the capsid coding regions of FMDV does not necessarily mean HS is required for efficient infection [20,22-24,34]. In the present study, the results from three different assays on two CHO cell lines are evidence for the HS-utilizing ability of O/Tibet/CHA/6/99tc, O/Fujian/CHA/9/99tc, rHN/TAR6-VP0 and rHN/FJ9-VP1, and the incapability of HS-dependent infection by the other eight viruses in WT-CHO cells (Figure 1, Figure 3A, Figure 4). Even so, a high affinity for heparin and the HS-binding of rHN, rHN/FJ9-VP0 and rHN/TAR6-VP0^Q2080L^ were supported by plaque reduction neutralization and inhibition assays on BHK-21 cells and virus adsorption assay on WT-CHO cells, respectively (Figure 3B, 3C; Figure 4). Loss of the heparin-sensitivity of these three viruses was accompanied by a single amino acid mutation Lys-1083 → Glu of VP1 (results not shown). It is also worth noting that, while heparin sensitivity of O/Tibet/CHA/6/99tc was detected in WT-CHO cells and the HS-binding and RGD integrin-binding sites of FMDV function independently of each other [32] (Figure 1, Figure 3A, Figure 4), the virus did not appear to initiate its HS-utilizing ability to facilitate viral infection in BHK-21 cells pre-incubated with the RGD-containing peptide VR-17 (Figure 3C). The distinct heparin affinity of O/Tibet/CHA/6/99tc, O/Fujian/CHA/9/99tc, rHN/TAR6-VP0 and rHN/FJ9-VP1 suggested that the specific amino acid residues on the outer capsid proteins (VP2 of O/Fujian/CHA/9/99tc and VP1 of rHN) may be related to FMDV-HS interaction on BHK-21 cells (Figure 3B, Table 1). As for the plaque phenotypes of all the selected PanAsia-1 strains and rescued viruses (Figure 1), the effect of a His or Tyr at residue 2079 of VP2 and a Lys or Glu at residue 1083 of VP1 on the local charged regions might contribute to the differences in the size of plaques produced by the corresponding viruses on BHK-21 cells (Table 1). Further work is needed to consider these two issues in more detail. One goal is to evaluate the cooperative effect of several amino acid residues in the capsid proteins of the HS-utilizing FMDVs and heparin-sensitive FMDVs on the adsorption efficiency and penetration capability of these viruses in WT-CHO cells. Another is to identify the type of heparin-sensitive cellular receptor(s) utilized by the heparin-sensitive FMDVs to facilitate viral infection in BHK-21 cells. Further studies will help us to interpret the positive selection of amino acid substitutions in the viral capsid for HS adaptation during FMDV microevolution in cultured cells.

In conclusion, our results demonstrate that VP2 of O/Tibet/CHA/6/99tc and VP1 of O/Fujian/CHA/9/99tc function as the critical regions in the acquisition of the HS-utilizing ability to infect WT-CHO cells. Gln-2080 → Leu in VP2 of O/Tibet/CHA/6/99tc and Lys-1083 → Glu in VP1 of O/Fujian/CHA/9/99tc would lead to the loss of the HS-utilizing ability of two chimeric viruses in WT-CHO cells, respectively. The distinct amino acid substitutions at the other positions in VP0 of O/Tibet/CHA/6/99tc and VP1 of O/Fujian/CHA/9/99tc may also play important roles in retaining the infectivity and the HS-utilizing capability of the corresponding site-directed mutant(s) in cultured cells. Although the HS-utilizing ability of O/Tibet/CHA/6/99tc is required to establish an efficient infection in WT-CHO cells, the virus was unable to utilize the cell surface HS for viral infection in BHK-21 cells. The heparin-sensitive FMDVs could bind to HS, but might be insufficient to initiate HS-dependent infection in WT-CHO cells.

## Methods

### Cell lines, plasmids and viruses

BHK-21 cells were maintained in Dulbecco’s modified Eagle’s medium (DMEM; Gibco) containing 10% fetal bovine serum (FBS; Hyclone) and 2 mM L-glutamine. WT-CHO cells (catalogue no. CCL-61) and HS-negative pgsD-677 cells (N-acetylglucosaminyl and glucuronyltransferase deficient, catalogue no. CRL-2244) were obtained from American type culture collection and were maintained in F-12 K nutrient mixture (Gibco) supplemented with 10% FBS and 100 U/mL penicillin-streptomycin. All cell lines were incubated at 37°C in a humidified chamber with 5% CO_2_.

The eukaryotic expression plasmid pCT7RNAP can express T7 RNA polymerase (T7 RNAP) in BHK-21 cells [41]. Plasmid pOFS containing the full-length infectious cDNA of FMDV O/HN/CHA/93 (Cathay topotype) under the control of the bacteriophage T7 promoter was constructed by Li *et al.*[42].

rHN is a genetically engineered virus recovered from the *Not* I-linearized pOFS *in vivo*[42]. Two bovine isolates of PanAsia-1 lineage of FMDV serotype O, O/Tibet/CHA/6/99wt and O/Fujian/CHA/9/99wt, were collected from Tibet Autonomous Region and FuJian province of China in 1999, respectively [43,44]. O/Tibet/CHA/6/99tc was derived from O/Tibet/CHA/6/99wt after 7 passages of BHK-21 cells. O/Fujian/CHA/9/99tc is a cell-adapted derivative of O/Fujian/CHA/9/99wt, which was persistently infected and serially passaged more than 26 times in BHK-21 cells.

### Construction of the chimeric and site-directed mutated full-length cDNA clones

The intra-serotype chimeric full-length cDNAs were generated by the exchange-cassette strategy to replace the individual VP0 and VP1 coding regions of an existing pOFS plasmid with the respective genes of O/Tibet/CHA/6/99tc and O/Fujian/CHA/9/99tc (Figure 2). Site-directed mutagenesis was performed by one-step overlap extension PCR with the Quick-Change® Multi Site-Directed Mutagenesis Kit (Stratagene) to produce four site-directed mutated full-length cDNAs with single amino acid substitutions at positions VP2-80 (exchange between Gln and Leu) and VP1-83 (exchange between Lys and Glu) in the corresponding chimeric full-length cDNAs, respectively (Figure 2). All the chimeric and site-directed mutated full-length genomic cDNA clones were confirmed by automated sequencing.

### Co-transfection and viral titrations

Plasmid constructions were linearized by digestion with *Not* I and subsequently co-transfected into subconfluent monolayers of BHK-21 cells (60% to 90% confluency) in 6-well plates with pCT7RNAP using the Lipofectamine 2000™ reagent (Invitrogen), according to the manufacturer’s instructions. The total RNAs were extracted from the harvested supernatants and the testing samples of four PanAsia-1 viruses, and the capsid coding regions were amplified by RT-PCR followed by sequencing. The supernatants of each rescued virus encoding the expected amino acid sequences of VP1–VP4 were stocked for further experiments.

### Studies of FMDV-HS interaction

Five different assays were performed with the proper concentrations of the specific FMDVs in BHK-21 cells (Integrin+, HS+), WT-CHO cells (Integrin-, HS+) and/or pgsD-677 cells (Integrin-, HS-), as described elsewhere [16,20,22,45].

### Plaque forming assay on BHK-21 cells and two CHO cell lines

BHK-21, WT-CHO and pgsD-677 cell monolayers in 6-well plates were inoculated with the serial ten-fold dilutions of all the selected PanAsia-1 strains and rescued viruses, following the previously described procedures [46]. The values of plaque titrations of each virus were calculated as described previously [24].

### Plaque reduction neutralization and inhibition assays on BHK-21 and WT-CHO cells

(i) **Plaque reduction neutralization assay on BHK-21 and WT-CHO cells.** The appropriate concentrations of the specific FMDVs (10–50 PFU; plaque forming units) were incubated with the amount (0–1 mg/mL; two-fold serial dilutions) of soluble heparin (Sigma; sodium salt) in phosphate-buffered saline (PBS, pH = 7.4) solution. After 10 min incubation at room temperature (RT) to allow heparin binding to FMDV, the reduction in virus titers of the mixtures was measured by plaque forming assay on BHK-21 cells and WT-CHO cells.

(ii) **Plaque inhibition assay on BHK-21 cells.** The RGD-containing peptide VR-17 (141-VPNLRGDLQVLAQKVAR-157 [47]; Invitrogen) was dissolved in PBS containing 1 mM CaCl_2_ and 0.5 mM MgCl_2_. Monolayers of BHK-21 cells were pre-incubated with the peptide VR-17 (0–1 mM; ten-fold serial dilutions) for 45 min prior to the addition of viruses for a further 1 h at 37°C. Next, 2 ml of the overlay medium containing 0.6% gum tragacanth were added to the cells. Finally, the cells were fixed with acetone/methanol (1:1) and stained with 0.2% crystal violet at 48 h post-infection. The inhibition of FMDV infection by the RGD-containing peptide VR-17 was calculated by PFU from the infected cell monolayers.

### Virus adsorption and penetration assays in WT-CHO cells

(i) **Virus adsorption assay in WT-CHO cells.** WT-CHO cells cultured on 24 mm coverslips in 6-well plates were incubated with the indicated FMDVs (50–100 PFU/cell) for 1 h at 4°C. Then, the inoculum was removed, the cells were washed with PBS, fixed with 4% paraformaldehyde and processed for an immunofluorescence assay (IFA) using a monoclonal antibody (Mab) 3E1 against FMDV serotype O (1/100) [48] and an Alexa Fluor® 594 goat anti-mouse immunoglobulin G (IgG) (1/400; Invitrogen).

(ii) **Virus penetration assay in WT-CHO cells.** In this assay, WT-CHO cells inoculated with the indicated viruses were washed with F-12 K at the end of the adsorption period, and fresh medium was added. The cells were then transferred to the normal incubation temperature (37°C). At the appropriate times post-infection (4 h), the cells were washed, fixed and examined for the expression of FMDV 3A protein with the primary antibodies (anti-3A Mab, 1/100) [49] and an Alexa Fluor® 488 goat anti-mouse immunoglobulin G (IgG) (1/400; Invitrogen).

The procedure of IFA was done as previously described [45,50,51]. Following IFA, the cells were washed with PBS and the nuclei were stained with DAPI (1/10,000; Beyotime) for 5 min at RT. Finally, the cells on coverslips were washed, mounted, and viewed with a Leica TCS SP8 confocal microscope.

## Competing interests

The authors declare that they have no competing interests.

## Authors’ contributions

XWB was involved in all the overall planning of the experiments and drafted the manuscript. HFB and PHL performed plaque assays and virus adsorption and penetration assays. WW generated the chimeric viruses and site-directed mutants. MZ carried out molecular modeling. PS contributed to reagents. YMC, ZJL and YFF participated in analysis of the data. BXX, YLC and DL delivered background information. JXL and ZXL conceived the study. All authors reviewed and approved the final manuscript.
